# Evaluation of Dynamic Changes and Regularity of Volatile Flavor Compounds for Different Green Plum (*Prunus mume* Sieb. et Zucc) Varieties during the Ripening Process by HS-GC–IMS with PLS-DA

**DOI:** 10.3390/foods12030551

**Published:** 2023-01-26

**Authors:** Haocheng Liu, Yuanshan Yu, Bo Zou, Yangyang Yu, Jiguo Yang, Yujuan Xu, Xiaowei Chen, Fan Yang

**Affiliations:** 1 Sericultural & Argi-Food Research Institute, Guangdong Academy of Agricultural Sciences/Key Laboratory of Functional Foods, Ministry of Agriculture and Rural Affairs/Guangdong Key Laboratory of Agricultural Products Processing, No. 133 Yiheng Street, Dongguanzhuang Road, Tianhe District, Guangzhou 510610, China; 2 School of Food Science and Engineering, South China University of Technology, Guangzhou 510641, China; 3 Liuliu Orchard Group Co., Ltd., Wuhu 241200, China

**Keywords:** green plums, gas chromatography–ion mobility spectrometry, flavor fingerprint, partial-least-squares discrimination analysis

## Abstract

Headspace gas chromatography–ion mobility spectrometry and partial-least-squares discriminant analysis (PLS-DA) were adopted to analyze the rule of change in flavor substances for different varieties of green plums at different levels of maturity (S1—immature, S2—commercially mature, and S3—fully mature). The results showed that 68 kinds of volatile flavor substances were identified in all green plum samples. The types and contents of such volatile substances experienced a V-shaped trend with an increasing degree of green plum maturity. During the S1 and S2 stages, aldehydes, ketones, and a small amount of alcohols were the main volatile flavor substances in the green plum samples. During the S3 stage, esters and alcohols were the most important volatile flavor components in the green plum pulp samples, followed by terpenes and ketones. YS had the most types and highest contents of volatile flavor substances in three stages, followed by GC and DZ. By using the PLS-DA method, this study revealed the differences in flavor of the different varieties of green plums at different maturity stages, and it identified eight common characteristic volatile flavor substances, such as ethyl acetate, 3-methylbutan-1-ol, and 2-propanone, produced by the different green plum samples during the ripening process, as well as the characteristic flavor substances of green plums at each maturity stage (S1–S3).

## 1. Introduction

Green plum (*Prunus mume* Sieb. et Zucc), also known as plum fruit and tart plum, is one of the most important crops of the Rosaceae family with stone fruits. Green plum is native to China and has been cultivated in China for 3000 years [1,2]. It is a traditional raw material for medicines and foods, and it has been described much in Chinese traditional culture (rich description in ancient literature) [3,4]. Nowadays, green plum is preferred by the public and popular in the market because of its rich nutrition and medicinal value [5].

Aroma is a key factor affecting the quality and consumer acceptance of green plum products [6]. Studying various aroma components will improve our understanding and help control key quality parameters that may affect green plum processing. The aroma substances in green plums are subject to many factors, such as varieties, cultivation conditions, climatic conditions, and maturity period [5,7]. Among them, the variety and maturity level are the main factors affecting the aroma of green plums. There is a large difference in the bioactive components of green plums of different varieties. Moreover, the difference resulting from the existence of various physiological, biochemical, and structural changes during the ripening process will naturally lead to green plums with different aroma properties [8,9]. This difference deserves a survey. In particular, key volatile flavor substances in green plums of different varieties at different maturity levels in China need to be identified and compared. However, previous studies focused on the identification of volatiles in a single variety of green plums [10,11,12]. Currently, scientific information on the aroma components and the rule of change for various green plum varieties and their maturity stages is limited.

For the analysis of fruit aroma substances, gas chromatography–mass spectrometry (GC–MS) and gas chromatography–olfactometry (GC–O-MS) are commonly used methods [13,14,15]. However, such methods require the pretreatment of samples, involving processes such as heating, distillation, and extraction, and have disadvantages such as cumbersome operations, extended detection time, excessive consumption of samples, and easy retention of organic solvents [16]. Furthermore, some original aroma components of fruits may be destroyed during the pretreatment process, leading to inaccurate measurement results [17]. Gas chromatography–ion mobility spectrometry (GC–IMS) is a new analytical technique that has been widely used for foods, traditional Chinese medicines, cosmetics, VOC monitoring, and other fields in recent years [18,19]. GC–IMS does not require any pretreatment and preparation of samples, thus helping to maintain the aroma of samples to the greatest extent. Compared with GC–MS, complex mixtures are first vaporized by a GC injector after separation in a column, and each neutral compound is transported to the ionization region of the IMS at different times, which facilitates tentative identification due to the retention times obtained from the GC. GC–IMS analyte data include both retention time and drift time, thus providing a two-dimensional chromatogram that increases the accuracy of the qualitative analysis. In addition, both GC and IMS are operated under atmospheric pressure, so the GC–IMS interface is simple and low-cost, making the miniaturization of its equipment possible [20,21]. When GC–IMS is used to analyze volatile substances, multivariate statistical methods are also usually adopted, such as modeling based on principal component analysis (PCA) and partial-least-squares discriminant analysis (PLS-DA). PCA can convert multiple indicators into several comprehensive indicators to extract properties and reveal relationships between variables [22]. PLS-DA based on PCA further inputs the converted score information into models to identify key contributors to the difference-related variables in the models [23,24]. As a result, it has been widely used in research on metabolomics and flavoromics [25,26].

This study used the GC–IMS technique to rapidly analyze and detect VOCs of seven Chinese superior green plum varieties at three maturity stages (immature, commercially mature, and fully mature) and to establish flavor fingerprints. On the basis of the analysis of the PLS-DA model, it then clarified the difference in volatile compounds in green plums of different varieties and at varied maturity levels. Finally, the variable importance in projection (VIP) was used to determine the key aroma substances in green plum samples of different varieties and at varied maturity levels. This paper aims to provide a theoretical basis for picking timing, storage, quality control, and subsequent utilization of green plum fruits.

## 2. Materials and Methods

### 2.1. Samples and Preparation

**Figure 1:**
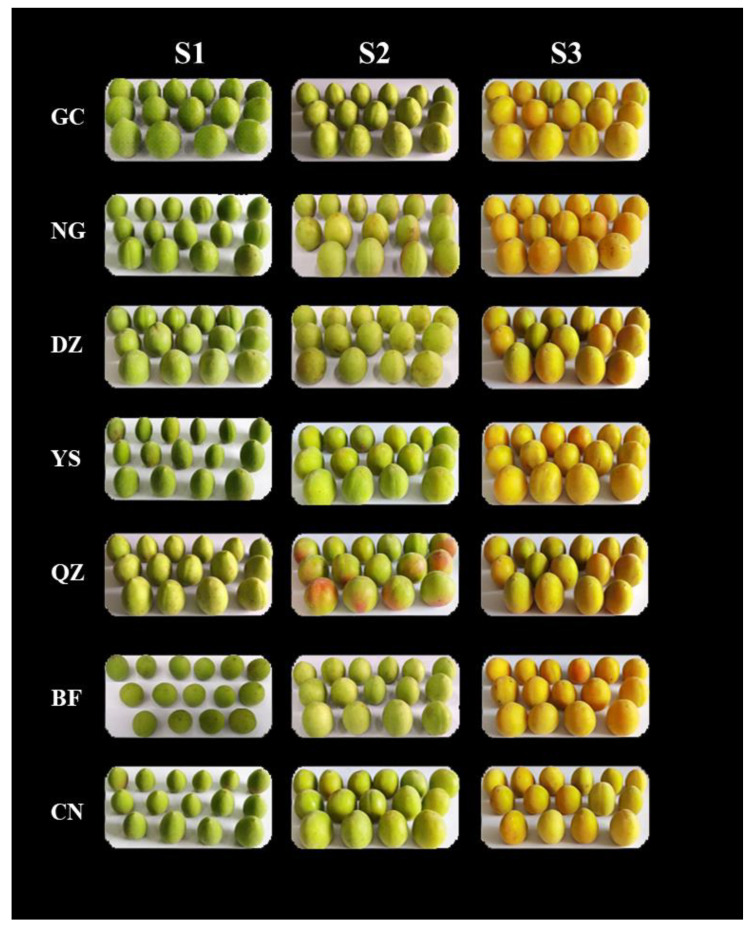
Seven representative and superior green plum varieties harvested at levels immature (S1), commercially mature (S2), and fully mature (S3).

Seven representative and superior green plum varieties were selected from the main producing areas of green plums in China: Qingzhu plum (QZ), Gaochan plum (GC), Nangao plum (NG), Dazhou plum (DZ), Baifen plum (BF), Changnong plum (CN), and Yingsu plum (YS). Details related to the cultivation locations of the seven cultivars are provided in supplementary data (Table S1). Green plums were uniform in size and were free from mechanical damage, diseases, and pests. Plums at three maturity levels, namely, immature (50–60%, S1), commercially mature (70–80%, S2), and fully mature (90–100%, S3), were picked as samples. A specific maturity stage was measured according to properties such as appearance and color (as shown in Figure 1). Immature green plum fruit was dark green. Commercially mature green plum was green or turquoise with occasional flushes. Fully mature green plum fruit was green–yellow or yellow. Skin color is an indicator of fruit ripeness and, as chlorophyll breaks down as the fruit ripens, the skin changes from green to yellow [14]. At each maturity stage, planting technicians from seven green-plum-producing areas randomly collected green plum fruits with the same appearance, color, and character from six green plum trees, where each tree was represented as a biological replicate. Ten fruits were grouped as one replicate. Three biological replicates were used for further experiments. After harvest, the fresh fruits were transported to the laboratory within two days, and all samples were washed, pitted, and pulped. The pulp was immediately frozen in liquid nitrogen and then stored at −80 °C for subsequent analysis.

### 2.2. HS-GC–IMS Analytical Methods

Samples were analyzed by a FlavourSpec^®^ GC–IMS. An Agilent 490 gas chromatograph (Agilent Technologies, Palo Alto, CA, USA) equipped with an FS-SE-54-CB capillary column (15 m × 0.53 mm) and IMS instrument was used for the analysis of green plum samples. An autosampler unit (CTC Analytics AG, Zwingen, Switzerland) was equipped so that samples could be directly inserted from the headspace using a sealed and heated 1 mL injector. After the thawing of green plum samples at 25 °C, accurate 2.0 g of the pulp was placed into a 20 mL headspace vial, and a 1 mL heated syringe used to automatically withdraw the sample headspace under the following analysis conditions:

The automatic injection conditions included an incubation temperature of 40 °C, 20 min incubation time, 500 rpm incubator speed, splitless mode, injection needle temperature of 85 °C, and 500 μL injection volume. The GC conditions included a 60 °C column temperature and a nitrogen carrier gas (purity ≥ 99.999%), with the flow rate set as follows: 2 mL/min for 2 min, 10 mL/min for 8 min, 100 mL/min for 10 min, and 150 mL/min for 10 min. The IMS conditions were as follows: 40 °C drift tube temperature and nitrogen (≥99.999%) as the drift gas at a flow rate of 150 mL/min. Each GC–IMS analysis was run in triplicate. To eliminate cross-contamination, the injector was purged with N_2_ flow for 30 s before each analysis and for 5 min after each analysis. C4–C9 *n*-ketones (Sinopharm Chemical Reagent Beijing Co. Ltd., Beijing, China) were used as an external reference to calculate the retention index (RI) of each substance. All volatile substances were identified by considering the RI and drift time of substances in the GC–IMS library [27].

### 2.3. Statistical Analysis

Laboratory Analytical Viewer (LAV), three plug-ins, and GC × IMS Library Search were adopted for the analysis of volatile substances. Qualitative analysis of volatile components was made by using NIST and IMS databases. At least three independent evaluations (*n* = 3) were performed to calculate the mean and standard deviation (SD) of the results. One-way ANOVA was performed by using SPSS 20.0 (SPSS Inc., Chicago, IL, USA). Origin 2016 (Origin Lab, Northampton, MA, USA) was used to draw the histogram-upset chart. MetaboAnalyst 5.0 (https://www.metaboanalyst.ca/, accessed on 7 May 2022) was used for partial-least-squares discriminant analysis (PLS-DA).

## 3. Results and Discussion

### 3.1. HS-GC–IMS Visual Topographic Plot Comparison

The HS-GC–IMS method was used to obtain all the information on volatile substances of the green plum samples to help identify the volatile substances and the rule of change in different green plum varieties during the ripening process. A difference comparison mode was used to show the differences between samples. The vertical and horizontal axes in the difference figure represent the retention time and ion migration time of volatile substances at the reactant ion peak, respectively, and each dot represents a volatile flavor substance or its dimers extracted from samples. The red area in the figure means that the concentration of the substance is higher than the reference value. The blue area shows that the concentration of the substance is lower than the reference value. The white area indicates that the concentration of the substance is equivalent to the reference value [28].

**Figure 2:**
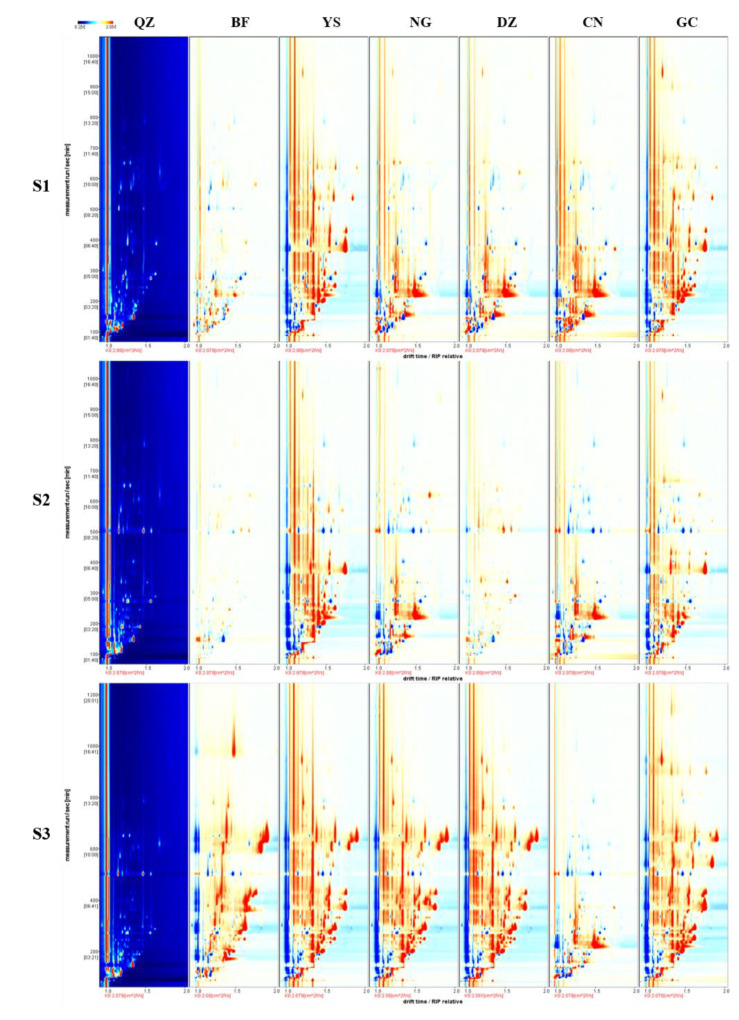
Two-dimensional chromatogram results of volatile fractional compositions in 7 varieties of green plum at three maturity stages.

With varieties and maturity levels as two dimensions for analysis, different varieties of green plums were selected as references to derive a plot of other samples (as shown in Figure 2). They clearly showed a difference in green plum pulp of different varieties at the three different maturity stages. The figure has a large area of dark-red and dark-blue dots, indicating that the quantity and concentration of volatile substances in green plums of different varieties experience significant changes during the three maturity stages. The derived plot of samples at each maturity stage as described above shows that the content of volatile flavor substances in green plum pulp varied remarkably with the maturity level. At the same time, for the pulp of green plums in the S3 stage, the number of red dots was significantly higher than that of blue dots, revealing that the content of most volatile flavor substances increased after the green plums had matured. We also observed similar results with the production of new volatile flavor substances in fruits [18]. Such a phenomenon is caused by the vigorous metabolism (metabolism of amino acids, fatty acids, carbohydrates, etc.) of mature fruits.

### 3.2. Identification of Substances

For the qualitative analysis of each volatile substance in the green-plum pulp samples, a comparison of the drift time and RI in IMS with those of true reference substances was made. Subsequently, 88 signal peaks (including monomers and dimers) from the 2D graph were confirmed, and a total of 68 volatile flavor substances was tentatively identified, as listed in Table 1. These include 23 kinds of esters, 16 kinds of alcohols, 12 kinds of ketones, 11 kinds of aldehydes, 3 kinds of terpenes, 2 kinds of heterocycles, and 1 kind of acid substance; these types basically cover all types of aroma substances in fruits [15,29,30]. Specifically, 20 kinds of substances such as amyl acetate, maltol, 2-heptanone, benzaldehyde, limonene, and 2-methylbutanoic acid formed dimers, which was mainly related to the concentration and proton affinity of volatile flavor substances. The protons of reactants of high-concentration substances with a proton affinity higher than that of water transferred to substances with a high proton affinity, thus contributing to the formation of dimers [18,31].

### 3.3. Analysis of VOC Fingerprints

**Figure 3:**
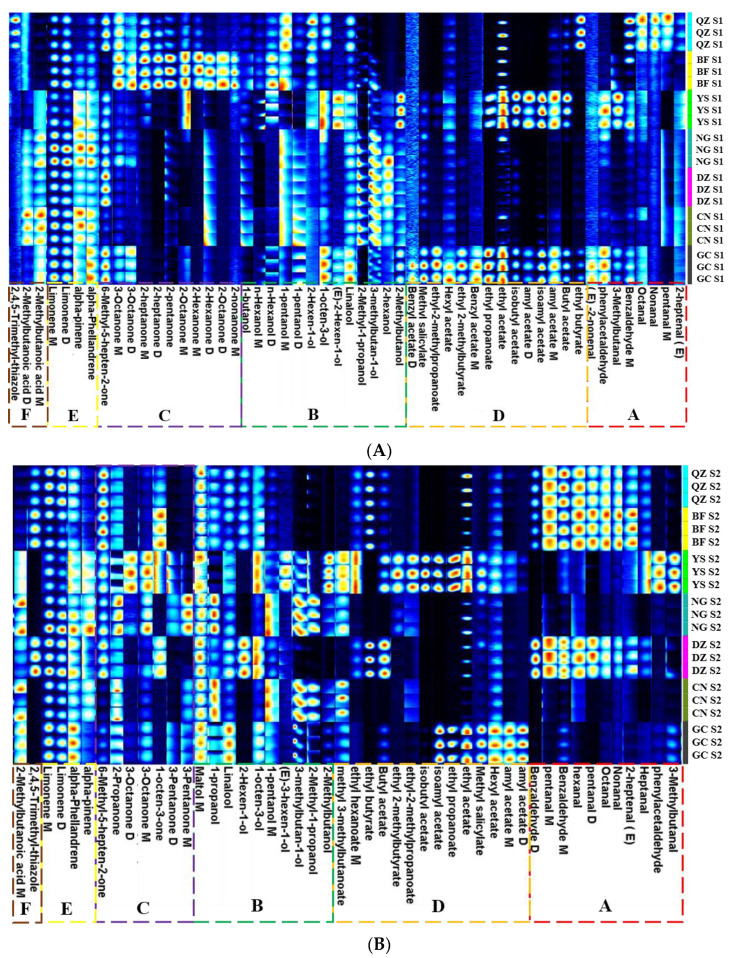

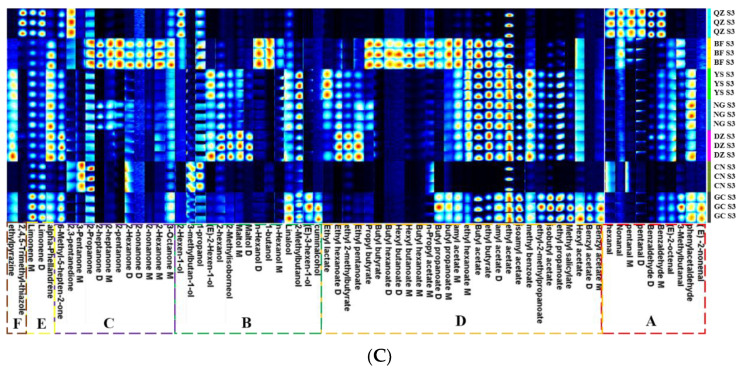
The VOC fingerprints of different green plum varieties of the same maturity: (**A**) S1 stages of green plum samples of 7 varieties; (**B**) S2 stages of green plum samples of 7 varieties; (**C**) S3 stages of green plum samples of 7 varieties.

Although the difference figure shows a trend of volatile components, it is difficult to accurately identify the properties of an individual substance in the figure. Using fingerprints is an ideal solution to this problem. A comparison of the spot intensities of VOCs at different stages can help determine the changes (increase, decrease, disappearance, or fluctuation) of volatile flavor substances in green plum pulp of different varieties and at varied maturity levels, as well as reveal dynamic changes of each substance [32]. In the fingerprint, each row represents an overall signal peak for one sample, and each column means the same substance in different samples. Each unit refers to the content of the substance at different moments. The color refers to the content of volatile substances. A brighter color means higher content. The green plum pulps of the three maturity stages of different varieties are shown in fingerprints in Figure 3A–C.

Six regions were divided according to the characteristic volatile substances of the fingerprint. For the green plum pulp in the S1 stage, the volatile substances of the three samples of NG, DZ, and CN were relatively close in composition and less volatile. Alcohols such as 2-methyl-1-propanol, 2-hexanol, and 3-methylbutan-1-ol, as well as 2-methylbutanoic acid, were their characteristic substances. The compositions of volatile substances in the two samples of YS and GC green plum seemed to be relatively close, and esters such as butyl acetate and ethyl acetate formed. After reaching the S2 stage, the compositions of volatile substances in the two samples of NG and CN were relatively close and less than those of other varieties. The compositions of volatile substances in the three samples QZ, BF, and DZ were relatively close. Their characteristic substances included six aldehydes, including benzaldehyde, (E)-2-heptenal, and hexanal, as well as 1-octen-3-one, 2,4,5-trimethyl-thiazole, 2-hexen-1-ol, and ethyl butyrate. At the same time, there were more esters in the pulp of YS and GC. In the S3 stage, the volatile substances in CN green plum pulp were the least among the seven green plums, and 3-methylbutan-1-ol and 3-pentanone were the characteristic components. The types of volatile substances in QZ green plum pulp were quite different and fewer than the other six, including four aldehydes such as hexanal and pentanal, as well as 2,3-pentanedione and 2,4,5-trimethyl-thiazole. 2-Hexen-1-ol was its characteristic volatile. Five kinds of green plum pulp (BF, YS, NG, DZ, and GC) released an abundance of esters, of which YS, NG, and DZ were the closest three. BF green plum pulp was quite different from them; its characteristic components included butyl butyrate, butyl hexanoate, hexyl butanoate, propyl acetate, n-hexanol, 1-butanol, 2-hexanone, and 2-nonanone.

### 3.4. Analysis of Dynamic Changes and Formation Regularity of VOCs

Further analysis in combination with Table 2 revealed that with an increase in maturity level, the esters in the seven kinds of green plums experienced a significant upward trend. Specifically, representative substances such as ethyl acetate, ethyl butyrate, isoamyl acetate, and isobutyl acetate were main contributors to the sweet and fruity aromas of the green plums [13]. This phenomenon resulted from the effect of alcohol acyltransferase during the ripening of green plums [16]. The content of ketones in green plum samples, except for the QZ sample, also had an upward trend with a rise in maturity level, which was caused by the enzymatic oxidation and degradation accelerated by carotenoids during the ripening of green plums [25]. The main substances produced included 6-methyl-5-hepten-2-one, 2-heptanone, 2-pentanone, and 2-propanone, and they had rich and complex aromas of violet, wood, and fruit [30]. In contrast, there was no obvious rule for the change in aldehydes, alcohols, terpenes, acids, and thiazoles. The contents and the rule of change of such substances in green plum samples of different varieties were different. Aldehydes are important contributors to the aroma of fresh green grass. With an increase in maturity level, the decrease in the concentration of aldehydes during the fruit ripening process may be a result of the combination with glutathione or the reduction of alcohol dehydrogenase [9]. This is consistent with the results of previous publications. The content of aldehydes in QZ, NG, and GC samples increased with a rise in maturity level. The main substances included benzaldehyde with aromas of hyacinths and almonds, as well as (E)-2-octenal and (E)-2-nonenal with cucumber aroma [33]. Alcohols serve as main contributors to the aroma of caramel and flowers [6] and result from the degradation of amino acids, carbohydrates, and lipids [34]. As the maturity level increased, the content of alcohols in BF, YS, and GC samples showed an upward trend, which was contrary to the trend of change in the content of aldehydes. Representative alcohols included 1-butanol, n-hexanol, and 3-methylbutan-1-ol with complex aromas of citrus fruit and brandy. Terpenoids are a kind of very important substances in fruits. They are probably generated by the metabolism of carbohydrates through isoprenoids pathway [27]. Except for the QZ and YS samples, the content of terpenoids in the other green plum samples decreased as the maturity level increased. Such terpenoids mainly included α-phellandrene, α-pinene, and limonene. During the growth of green plums, the content of 2-methylbutanoic acid showed a downward trend, while the content of 2,4,5-trimethyl-thiazole and ethyl pyrazine with complex aromas of chocolates and roast beef experienced an upward trend.

The above findings revealed that with the improvement of maturity, the volatile substances and contents of green plum samples first decreased and then increased. During S1 and S2, green plum samples mainly consisted of aldehydes and ketones, while during S3, esters and alcohols were the most important volatile flavor components in the green plum pulp sample, followed by terpenes, ketones, and others (acids, heterocycles). The content of volatile substances in the S3 stage reached the maximum value. The S2 stage is the continuous accumulation stage of fruit free amino acids, carotenoids, glycosides, unsaturated fatty acids, and other aroma precursor substances. Here, the activity decreases, and volatile substances show a downward trend [14]. After the accumulation reaches a certain stage, the metabolism is vigorous, and various aromas develop [25]. The degradation rate of precursor substances through hydrolysis, redox reactions, and other pathways significantly increases, resulting in an increase in the content of volatile substances [34].

**Figure 4:**
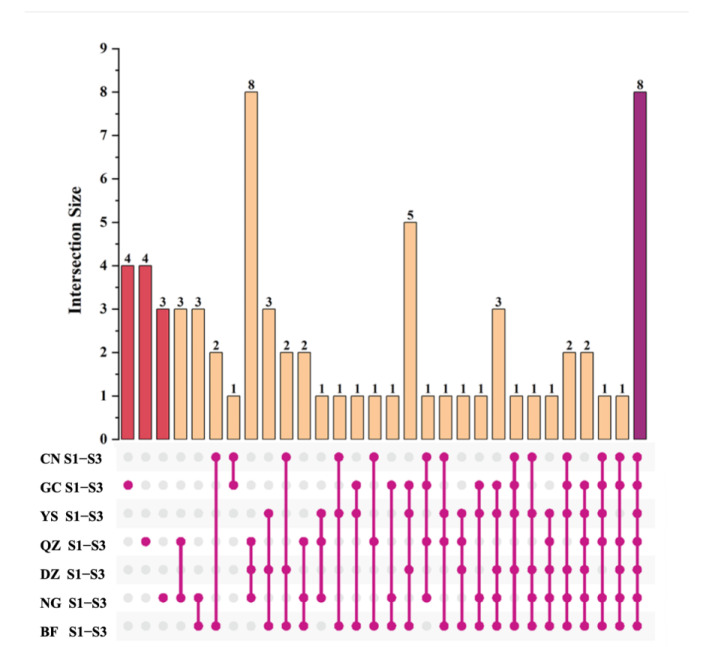
Upset plots of volatiles during three ripening periods for seven green plum varieties. Histogram: Red indicates the amount of unique volatile substances in GC, QZ, and NG green plums; purple indicates the amount of volatile substances in common among the 7 green plum samples; yellow indicates the amount of volatile substances in common among the 2/3/4/5/6 green plum samples.

At the same time, the peak intensities of the fingerprints were integrated; these were classified as shown in Figure 4. We found that eight substances, namely, benzaldehyde, 3-methylbutan-1-ol, 6-methyl-5-hepten-2-one, ethyl acetate, ethyl propanoate, α-phellandrene, and limonene D/M are volatile flavor substances shared by green plums; these endow green plums with common aroma properties. In addition, BF, GC, and QZ green plum samples contain unique volatile substances, which are butyl butyrate, hexyl butanoate, and n-propyl acetate in BF; (E)-2-nonenal, (E)-3-hexen-1-ol, cumin alcohol, and benzyl acetate in GC; and heptanal, 1-propanol, 2,3-pentanedione, and cyclohexanone in QZ. Analysis of Table 2 also showed that the content of aroma substances in the YS green plum sample at the three maturity stages was higher than that of the other green plum varieties. As the fruit maturity level increased, there was an increase in the content of volatile aroma components such as esters, ketones, alcohols, terpenes, and acids, as well as a slight decrease in the content of aldehydes. Hence, the YS green plum sample had stronger fruity and floral aromas as well as caramel and citrus aromas than those of the other samples. YS green plum had the highest aroma quality, followed by the NG, GC, DZ, BF, QZ, and CN green plum samples.

### 3.5. PLS-DA-Based Fingerprint

PLS-DA, a supervised discriminant analysis method, can identify complex and difficult-to-find variables, as well as assess the regularity of and differences between samples [23,35]. In this experiment, a model of the correlation between the peak intensities of VOCs measured by HS-GC–IMS and sample types at the three maturation stages was built to identify the differences in volatile substances in different maturity stages. The VIP method was used to explore characteristic aromas in the PLS-DA model. The overall sample size in the model was 21 (7 varieties × 3 times). The Y variable in the three models referred to seven kinds of green plum pulp in three maturity stages (immature, commercially mature, and fully mature). The X variable referred to 68 VOCs identified by HS-GC–IMS.

**Figure 5:**
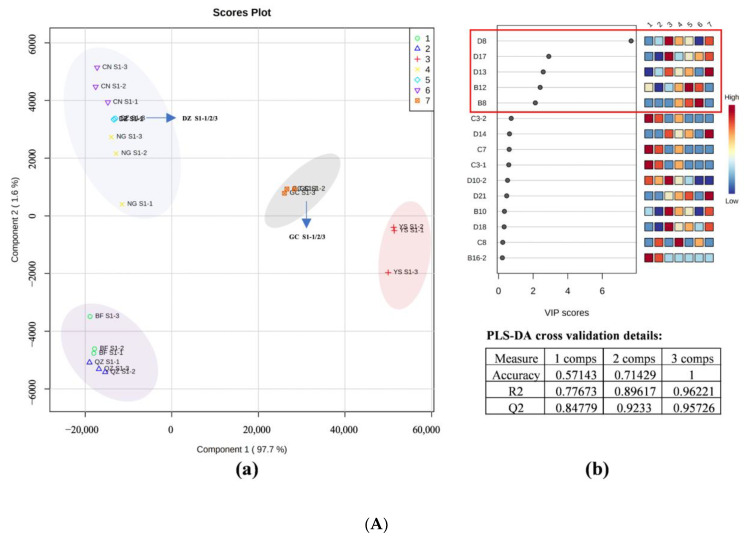

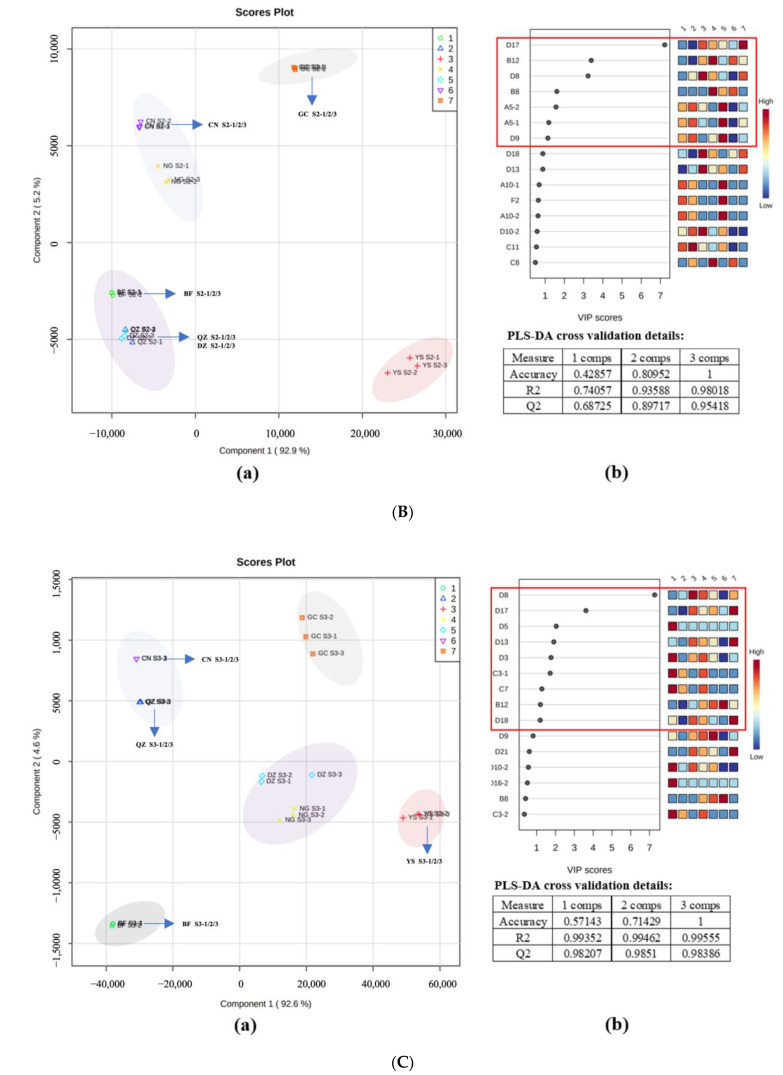
Partial least squares-discriminant analysis (PLS-DA) of gas chromatography–ion mobility spectrometry (GC–IMS) data. (**A**) S1 stages of green plum samples of 7 varieties; (**B**) S2 stages of green plum samples of 7 varieties; (**C**) S3 stages of green plum samples of 7 varieties. The figure includes: (a) PLS-DA score plot; (b) variable importance in projection (VIP) scores of each variable and cross-validation results.

According to the HS-GC–IMS PLS-DA score plot, the first two components in the models for S1, S2, and S3 maturity stages respectively accounted for 99.3%, 98.1%, and 97.2% of the total variables. Moreover, parameters for statistics and validation, such as accuracy, goodness of fit (R2), and goodness of prediction (Q2), were used to compare the performance of these PLS-DA models. Accuracy, R2, and Q2 were all higher than 0.95 in the three models. This indicated that the three models were accurate and robust [24]. The PLS-DA score plot could directly reflect the similarity and difference between samples. The greater difference between two samples represented a farther distance between locations in the score plot, and vice versa [36]. According to the PLS-DA score plot of the samples in Figure 5A–C, seven groups of samples were well-separated from each other in three models, indicating that the HS-GC–IMS method for the collection and detection of volatiles is proper for the classification of green plums. The distinguishing effects of these models are shown in Figure 5A–C. In the S1 stage, the aromas of QZ and BF are close; the aromas of NG, CN, and DZ are close; and the aromas of YS and GC are close. In the S2 stage, NG and CN are also close in aroma, DZ is close to QZ and BF in aroma, YS and GC are also relatively independent, and the aroma is unique. In the S3 stage after the fruit is fully mature, the aromas of DZ and NG are close, the aromas of QZ and CN are close, and BF, YS, and GC are relatively independent. This result is consistent with the fingerprint analysis of GC–IMS, indicating that PLS-DA is an effective method for distinguishing green plum samples of different maturities by volatile-flavor characteristics.

The influence on and explanatory power of each variable for the classification and identification of each group of samples were assessed by calculating VIP [37,38]. A higher value of VIP meant a greater difference in aroma components between groups and was more important for the classification of aromas of green plums. According to the VIP score plot for the S1–S3 stages, the VIP values of 5, 7, and 9 (including dimers) volatile aroma substances in the respective stage were higher than 1, indicating that these VOCs were key characteristic flavor substances of the different green plum varieties in the three maturity stages. Among them, the VIP values of ethyl acetate, isoamyl acetate, and 3-methylbutan-1-ol were greater than 1 in all maturation stages; they are representative aroma substances belonging to green plums during the ripening process. The marker flavors in the S1 stage (VIP > 1) are ethyl propanoate and 2-methyl-1-propanol. The S2 stage has benzaldehyde and ethyl butyrate; and the S3 stage has 2-heptanone, 2-pentanone, butyl acetate, butyl butyrate, ethyl propanoate, and isobutyl acetate.

## 4. Conclusions

In this research, the changes in flavor substances of seven varieties of green plums at three maturity stages were measured by using the HS-GC–IMS technique. The GC–IMS results showed that 88 signal peaks and 68 volatile flavor substances were identified in the green plum samples. The flavor fingerprint screened eight kinds of key flavor substances common to green plums and found that BF, GC, and QZ green plum samples contained unique volatile substances. The content of volatile substances in green plum samples experienced a V-shaped trend with an increasing degree of green plum maturity, and the content of volatile substances in the S3 stage reached the maximum value. The content of volatile substances in all green plums reached the highest level in the S3 stage. Specifically, with a rise in maturity level, the content of esters and ketones showed a significant upward trend, while the content of acids decreased. For other kinds of substances, there was no obvious rule of change. Such findings confirm that the formation of aromas of green plum fruit is a dynamic process. The YS green plum sample had stronger fruity and floral aromas as well as caramel and citrus aromas than those of the other samples, and it had the highest aroma quality, followed by GC and DZ. By using the PLS-DA model, researchers effectively classified different green plum varieties at different maturity levels. They also identified three key characteristic volatile flavor substances in the different green plum varieties during the ripening process and characteristic substances in each stage (S1–S3). Fingerprints were created by using HS-GC–IMS and PLS-DA. This is a simple, specific, and reliable method for assessing the characteristic volatile substances in green plum samples. It requires a minimum of sample preparation steps and reduces the time necessary for analysis. Considering such advantages, a combination of HS-GC–IMS-based fingerprints and PLS-DA could identify and classify the maturity level of green plum samples, which theoretically and technically supported the quality stability maintenance and identification of green plum processed in maturity levels.

## Figures and Tables

**Table 1:** Volatile compounds identified in three maturation stages of different green plums using GC–IMS.

Count	Compounds	CAS#	Formula	MW	RI ^a^	Rt ^b^	Dt ^c^	Identification Approach
Aldehydes								
A1	(E)-2-nonenal	18829-56-6	C_9_H_16_O	140.2	1167	901.514	1.40456	RI, Dt
A2	(E)-2-octenal	2548-87-0	C_8_H_14_O	126.2	1035	655.16	1.82104	RI, Dt
A3	(E)-2-heptenal	18829-55-5	C_7_H_12_O	112.2	954.3	504.555	1.25426	RI, Dt
A4	3-methylbutanal	590-86-3	C_5_H_10_O	86.1	658.7	173.618	1.41089	RI, Dt
A5-1	benzaldehyde M ^d^	100-52-7	C_7_H_6_O	106.1	955	505.944	1.46953	RI, Dt
A5-2	benzaldehyde D ^e^	100-52-7	C_7_H_6_O	106.1	955	505.944	1.14839	RI, Dt
A6	heptanal	111-71-7	C_7_H_14_O	114.2	899.8	403.046	1.33324	RI, Dt
A7	hexanal	66-25-1	C_6_H_12_O	100.2	790.4	273.455	1.25514	RI, Dt
A8	nonanal	124-19-6	C_9_H_18_O	142.2	1106.1	787.759	1.47369	RI, Dt
A9	octanal	124-13-0	C_8_H_16_O	128.2	1009.9	608.339	1.40676	RI, Dt
A10-1	pentanal D	110-62-3	C_5_H_10_O	86.1	694.1	191.548	1.42316	RI, Dt
A10-2	pentanal M	110-62-3	C_5_H_10_O	86.1	694.5	191.892	1.18296	RI, Dt
A11	phenylacetaldehyde	122-78-1	C_8_H_8_O	120.2	991.4	573.768	1.53538	RI, Dt
Alcohols								
B1	(E)-2-hexen-1-ol	928-95-0	C_6_H_12_O	100.2	902.3	407.734	1.51487	RI, Dt
B2	1-butanol	71-36-3	C_4_H_10_O	74.1	657	172.876	1.37972	RI, Dt
B3	(E)-3-hexen-1-ol	928-97-2	C_6_H_12_O	100.2	864.6	357.634	1.52914	RI, Dt
B4	1-octen-3-ol	3391-86-4	C_8_H_16_O	128.2	982.5	557.24	1.15661	RI, Dt
B5-1	1-pentanol D	71-41-0	C_5_H_12_O	88.1	759.1	245.531	1.50921	RI, Dt
B5-2	1-pentanol M	71-41-0	C_5_H_12_O	88.1	761.6	247.643	1.25143	RI, Dt
B6	1-propanol	71-23-8	C_3_H_8_O	60.1	580.1	138.542	1.11011	RI, Dt
B7	2-hexanol	626-93-7	C_6_H_14_O	102.2	768.4	253.22	1.5696	RI, Dt
B8	2-methyl-1-propanol	78-83-1	C_4_H_10_O	74.1	621	156.782	1.36365	RI, Dt
B9	2-hexen-1-ol	2305-21-7	C_6_H_12_O	100.2	849.3	340.218	1.17979	RI, Dt
B10	2-methylbutanol	137-32-6	C_5_H_12_O	88.1	766.1	251.358	1.47465	RI, Dt
B11	2-methylisoborneol	2371-42-8	C_11_H_20_O	168.3	1171.1	909.235	1.26369	RI, Dt
B12	3-methylbutan-1-ol	123-51-3	C_5_H_12_O	88.1	731	222.234	1.48826	RI, Dt
B13	cumin alcohol	536-60-7	C_10_H_14_O	150.2	1296	1142.267	1.32661	RI, Dt
B14	linalool	78-70-6	C_10_H_18_O	154.3	1062.1	705.745	1.21707	RI, Dt
B15-1	maltol D	118-71-8	C_6_H_6_O_3_	126.1	1092.3	762.012	1.60266	RI, Dt
B15-2	maltol M	118-71-8	C_6_H_6_O_3_	126.1	1091.5	760.659	1.21276	RI, Dt
B16-1	n-hexanol D	111-27-3	C_6_H_14_O	102.2	868	361.45	1.63935	RI, Dt
B16-2	n-hexanol M	111-27-3	C_6_H_14_O	102.2	868.7	362.253	1.32543	RI, Dt
Ketones								
C1	1-octen-3-one	4312-99-6	C_8_H_14_O	126.2	979.5	551.692	1.2718	RI, Dt
C2	2,3-pentanedione	600-14-6	C_5_H_8_O_2_	100.1	705.7	201.203	1.21544	RI, Dt
C3-1	2-heptanone D	110-43-0	C_7_H_14_O	114.2	893.1	390.601	1.63177	RI, Dt
C3-2	2-heptanone M	110-43-0	C_7_H_14_O	114.2	894.3	392.798	1.26173	RI, Dt
C4-1	2-hexanone M	591-78-6	C_6_H_12_O	100.2	784.3	266.503	1.18638	RI, Dt
C4-2	2-hexanone D	591-78-6	C_6_H_12_O	100.2	781.9	264.491	1.50278	RI, Dt
C5-1	2-nonanone D	821-55-6	C_9_H_18_O	142.2	1096.9	770.615	1.87878	RI, Dt
C5-2	2-nonanone M	821-55-6	C_9_H_18_O	142.2	1100.4	777.192	1.40774	RI, Dt
C6-1	2-octanone D	111-13-7	C_8_H_16_O	128.2	997.1	584.423	1.75543	RI, Dt
C6-2	2-octanone M	111-13-7	C_8_H_16_O	128.2	997.3	584.748	1.33435	RI, Dt
C7	2-pentanone	107-87-9	C_5_H_10_O	86.1	686.8	186.181	1.37047	RI, Dt
C8	2-propanone	67-64-1	C_3_H_6_O	58.1	520.3	111.855	1.1139	RI, Dt
C9-1	3-octanone D	106-68-3	C_8_H_16_O	128.2	990.7	572.555	1.71809	RI, Dt
C9-2	3-octanone M	106-68-3	C_8_H_16_O	128.2	991.2	573.358	1.30758	RI, Dt
C10-1	3-pentanone D	96-22-0	C_5_H_10_O	86.1	694	191.444	1.35447	RI, Dt
C10-2	3-pentanone M	96-22-0	C_5_H_10_O	86.1	696	193.097	1.10848	RI, Dt
C11	6-methyl-5-hepten-2-one	110-93-0	C_8_H_14_O	126.2	992.4	575.635	1.17731	RI, Dt
C12	cyclohexanone	108-94-1	C_6_H_10_O	98.1	896.4	396.71	1.15243	RI, Dt
Esters								
D1-1	amyl acetate D	628-63-7	C_7_H_14_O_2_	130.2	916.1	433.442	1.764	RI, Dt
D1-2	amyl acetate M	628-63-7	C_7_H_14_O_2_	130.2	916.7	434.541	1.31285	RI, Dt
D2-1	benzyl acetate D	140-11-4	C_9_H_10_O_2_	150.2	1168.9	904.987	1.76778	RI, Dt
D2-2	benzyl acetate M	140-11-4	C_9_H_10_O_2_	150.2	1168.2	903.709	1.32639	RI, Dt
D3	butyl acetate	123-86-4	C_6_H_12_O_2_	116.2	804.6	289.538	1.61954	RI, Dt
D4	ethyl 2-methylbutyrate	7452-79-1	C_7_H_14_O_2_	130.2	844	334.251	1.65277	RI, Dt
D5	butyl butyrate	109-21-7	C_8_H_16_O_2_	144.2	1009.9	608.224	1.82457	RI, Dt
D6-1	butyl hexanoate D	626-82-4	C_10_H_20_O_2_	172.3	1209.6	980.988	2.05481	RI, Dt
D6-2	butyl hexanoate M	626-82-4	C_10_H_20_O_2_	172.3	1208.8	979.538	1.46908	RI, Dt
D7-1	butyl propanoate D	590-01-2	C_7_H_14_O_2_	130.2	909.3	420.78	1.72179	RI, Dt
D7-2	butyl propanoate M	590-01-2	C_7_H_14_O_2_	130.2	909.6	421.354	1.2867	RI, Dt
D8	ethyl acetate	141-78-6	C_4_H_8_O_2_	88.1	605.8	150.027	1.33841	RI, Dt
D9	ethyl butyrate	105-54-4	C_6_H_12_O_2_	116.2	793	276.356	1.56065	RI, Dt
D10-1	ethyl hexanoate D	123-66-0	C_8_H_16_O_2_	144.2	1009.2	607.007	1.79623	RI, Dt
D10-2	ethyl hexanoate M	123-66-0	C_8_H_16_O_2_	144.2	1011	610.302	1.34063	RI, Dt
D11	ethyl lactate	97-64-3	C_5_H_10_O_3_	118.1	844.9	335.177	1.53478	RI, Dt
D12	ethyl pentanoate	539-82-2	C_7_H_14_O_2_	130.2	901.3	405.905	1.68137	RI, Dt
D13	ethyl propanoate	105-37-3	C_5_H_10_O_2_	102.1	705.4	200.94	1.45255	RI, Dt
D14	ethyl-2-methylpropanoate	97-62-1	C_6_H_12_O_2_	116.2	750.6	238.502	1.56531	RI, Dt
D15	hexyl acetate	142-92-7	C_8_H_16_O_2_	144.2	1042.9	669.943	1.41084	RI, Dt
D16-1	hexyl butanoate D	2639-63-6	C_10_H_20_O_2_	172.3	1210.2	982.075	2.0827	RI, Dt
D16-2	hexyl butanoate M	2639-63-6	C_10_H_20_O_2_	172.3	1207.8	977.727	1.48683	RI, Dt
D17	isoamyl acetate	123-92-2	C_7_H_14_O_2_	130.2	877.3	371.926	1.74845	RI, Dt
D18	isobutyl acetate	110-19-0	C_6_H_12_O_2_	116.2	768.3	253.199	1.6155	RI, Dt
D19	methyl 3-methylbutanoate	556-24-1	C_6_H_12_O_2_	116.2	765.8	251.078	1.53391	RI, Dt
D20	methyl benzoate	93-58-3	C_8_H_8_O_2_	136.1	1054.9	692.207	1.20739	RI, Dt
D21	methyl salicylate	119-36-8	C_8_H_8_O_3_	152.1	1192.8	949.699	1.19979	RI, Dt
D22-1	n-propyl acetate D	109-60-4	C_5_H_10_O_2_	102.1	709	203.911	1.47959	RI, Dt
D22-2	n-propyl acetate M	109-60-4	C_5_H_10_O_2_	102.1	709.7	204.539	1.16613	RI, Dt
D23	propyl butyrate	105-66-8	C_7_H_14_O_2_	130.2	915.3	432.035	1.68986	RI, Dt
Terpenes								
E1	α-phellandrene	99-83-2	C_10_H_16_	136.2	993.6	577.939	1.21878	RI, Dt
E2	α-pinene	80-56-8	C_10_H_16_	136.2	974	541.289	1.21564	RI, Dt
E3-1	limonene D	138-86-3	C_10_H_16_	136.2	1033.9	653.05	1.29495	RI, Dt
E3-2	limonene M	138-86-3	C_10_H_16_	136.2	1034.4	653.972	1.21703	RI, Dt
Others								
F1-1	2-methylbutanoic acid D	116-53-0	C_5_H_10_O_2_	102.1	844.5	334.793	1.47419	RI, Dt
F1-2	2-methylbutanoic acid M	116-53-0	C_5_H_10_O_2_	102.1	846.8	337.429	1.20282	RI, Dt
F2	2,4,5-trimethyl-thiazole	13623-11-5	C_6_H_9_NS	127.2	955.3	506.511	1.56675	RI, Dt
F3	ethyl pyrazine	13925-00-3	C_6_H_8_N_2_	108.1	923.3	446.836	1.5138	RI, Dt

^a^ Represents the retention index calculated using n-ketones C4–C9 as external standard on FS-SE-54-CB column. ^b^ Represents the retention time in the capillary GC column. ^c^ Represents the drift time in the drift tube. ^d^ Represents the monomer of the substance. ^e^ Represents the dimer of the substance.

**Table 2:** Peak intensities and relative contents of volatile substances in different green plum samples during S1–S3.

Cultivar	Aldehydes	Alcohols	Ketones	Esters	Terpenes	Others	Total
	Peak Intensity						
BF S1	985.34 ± 86.33	3043.29 ± 184.72	5919.63 ± 398.66	8236.98 ± 313.37	1026.19 ± 98.42	61.57 ± 1.76	19,273 ± 1083.26
BF S2	6674.48 ± 136.04	1200.21 ± 43.6	1385.02 ± 130.58	5229.23 ± 573.34	828.76 ± 216.49	918.07 ± 2.84	16,235.77 ± 1102.89
BF S3	1361.29 ± 174.67	4641.56 ± 89.67	17,825.5 ± 552.55	59,926.1 ± 920.71	544.62 ± 84.93	80.63 ± 6.33	84,379.7 ± 1828.86
QZ S1	2042.13 ± 373.82	2163.97 ± 137.18	4652.09 ± 372.82	10,210.29 ± 357.07	840.7 ± 97.3	96.2 ± 7.22	20,005.38 ± 1345.41
QZ S2	6963.52 ± 247.39	1372.2 ± 95.86	1858.84 ± 114.55	10,271.08 ± 1394.71	1185.12 ± 19.29	564.42 ± 18.47	22,215.18 ± 1890.27
QZ S3	5842.44 ± 132.97	1442.62 ± 88.76	1907.61 ± 81.35	13,676.02 ± 336.03	1030.24 ± 68.82	436.16 ± 13.98	24,335.09 ± 721.91
YS S1	1995.28 ± 276.7	5195.08 ± 61.51	503.05 ± 18.55	114,268.68 ± 502.48	684.4 ± 127.63	63.62 ± 2.32	122,710.11 ± 989.19
YS S2	1581.34 ± 56.45	5936.45 ± 70.78	788.69 ± 32.07	97,161.86 ± 524.26	708.84 ± 60.52	26.57 ± 3.09	106,203.75 ± 747.17
YS S3	1766.35 ± 124.74	5548.3 ± 89.53	679.17 ± 42.73	165,322.85 ± 506.43	790.83 ± 11.66	151.64 ± 3.61	174,259.14 ± 778.7
NG S1	458.45 ± 43.88	11,997.74 ± 251.77	3937.94 ± 479.05	15,616.84 ± 864.18	1398.19 ± 203.08	168.74 ± 12.13	33,577.9 ± 1854.09
NG S2	326.46 ± 19.18	10,800.05 ± 115.55	3211.11 ± 239.55	11,227.39 ± 199.9	1188.26 ± 243.21	148.41 ± 30.08	26,901.68 ± 847.47
NG S3	1707.45 ± 124.88	6174.68 ± 209.84	10,698.35 ± 731.89	116,221.99 ± 509.95	919.1 ± 54.81	232.98 ± 5.02	135,954.55 ± 1636.39
DZ S1	821.23 ± 100.06	13,209.91 ± 113.87	1082.07 ± 22.78	10,511.61 ± 410.34	992.94 ± 10.09	63.6 ± 18.15	26,681.36 ± 675.29
DZ S2	9955.28 ± 396.93	1947.84 ± 476.79	659.41 ± 28.73	11,251.03 ± 690.35	1163.73 ± 142	1071.24 ± 166.37	26,048.53 ± 1901.17
DZ S3	1837.69 ± 564.91	7150.64 ± 401.4	1168.35 ± 80.86	110,524.83 ± 1369.02	774.07 ± 33.61	224.84 ± 65.65	121,680.42 ± 2515.45
CN S1	139.45 ± 47.07	14,392.84 ± 122.98	1217.91 ± 112.41	5936.16 ± 255.15	1181.34 ± 133.07	532.22 ± 68.14	23,399.92 ± 738.82
CN S2	113.13 ± 20.69	9834.38 ± 50.79	2028.3 ± 272.07	2011.28 ± 69.95	939.72 ± 16.36	303.05 ± 69.78	15,229.86 ± 499.64
CN S3	72.01 ± 6.57	9786.64 ± 22.18	2827.46 ± 55.88	10,245.83 ± 32.53	713.37 ± 20.72	185.3 ± 14.54	23,830.61 ± 152.42
GC S1	1679.03 ± 239.49	3989.75 ± 186.25	878.52 ± 44.99	82,941.45 ± 293.88	1276.94 ± 97.44	0	90,765.69 ± 862.05
GC S2	1093.02 ± 65.17	2142.51 ± 68.9	328.1 ± 14.1	51,224.58 ± 427.59	1253.39 ± 7.97	0	56,041.6 ± 583.73
GC S3	2421.12 ± 431.3	4907.02 ± 419.92	1035.85 ± 15.24	119,377.73 ± 836.67	1238.32 ± 112.33	0	128,980.04 ± 1815.46

## Data Availability

Data are contained within the article or supplementary material.

## Supplementary Materials

The following supporting information can be downloaded at: https://www.mdpi.com/article/10.3390/foods12030551/s1, Supplementary data (Table S1. Information on cultivation sites of 7 kinds of green plum).
